# Filgrastim as a Rescue Therapy for Persistent Neutropenia in a Case of Dengue Hemorrhagic Fever with Acute Respiratory Distress Syndrome and Myocarditis

**DOI:** 10.1155/2011/896783

**Published:** 2011-11-10

**Authors:** Desh Deepak, Rakesh Garg, Mridula Pawar, Neerja Banerjee, Rakesh Solanki, Indubala Maurya

**Affiliations:** Department of Anaesthesiology and Intensive Care, Postgraduate Institute of Medical Education and Research and Dr Ram Manohar Lohia Hospital, New Delhi 10041, India

## Abstract

Pathogenesis of dengue involves suppression of immune system leading to development of characteristic presentation of haematological picture of thrombocytopenia and leucopenia. Sometimes, this suppression in immune response is responsible for deterioration in clinical status of the patient in spite of all specific and supportive therapy. Certain drugs like steroids are used for rescue therapy in conditions like sepsis. We present a novel use of filgrastim as a rescue therapy in a patient with dengue hemorrhagic fever (DHF) with acute respiratory distress syndrome (ARDS), myocarditis, and febrile neutropenia and not responding to standard management.

## 1. Introduction

The management of dengue hemorrhagic shock with ongoing deterioration in clinical status like multiorgan failure is a real challenge for an intensivist. Pathophysiologic mechanisms have not been well described in dengue, although immune suppression has been cited leading to thrombocytopenia and leucopenia [1, 2]. Filgrastim, a granulocyte colony-stimulating factor (G-CSF) has been used as adjuvant therapy in patients of pneumonia with sepsis [3]. We hereby report a case of dengue hemorrhagic fever (DHF) with acute respiratory distress syndrome (ARDS), myocarditis, and febrile neutropenia which improved with the usage of filgrastim as a rescue therapy.

## 2. Case Report

A 23-year-old female presented to medical clinics with history of cough, sore throat, and high grade fever for last 7 days. The fever was high grade (101–103°F) and was not associated with chills and rigor. On reviewing the history, she did not have any other comorbid illness. On investigations, she was found to have positive serology for dengue and her platelet count was 20,000/mm^3^. She was admitted in the medical ward, and 6 hours later her blood pressure dropped to 82/42 mm Hg. After adequately resuscitating her with intravenous fluids, she was started on dopamine infusion and titrated at 5 *μ*g/kg/min to maintain her blood pressure at 106/62 mm Hg. She was shifted to intensive care unit for further management. The patient's respiratory rate increased to 35/minute. Her chest X-ray revealed bilateral pulmonary infiltrates suggestive of acute respiratory distress syndrome. Her oxygen saturation (SpO_2_) was 88% on oxygen by face mask. Arterial blood gas analysis revealed a paO_2_ of 55 mm Hg. She was given a trial of noninvasive ventilation but she could not maintain oxygen saturation (SpO_2_—84–86%). She was tracheally intubated and started on mechanical ventilation. Her repeat platelet count was 12,000/mm^3^. Other laboratory investigations revealed haemoglobin 11.8 g/dL, total leucocyte count (TLC): 10,800/mm^3^, differential leucocyte counts: neutrophil: 85%, lymphocytes: 13%, sodium: 144 mg/L, potassium: 3.6 mg/L, blood urea: 48 mg/dL, serum creatinine: 1.1 mg/dL and total bilirubin: 0.9 mg/dL. Her international normalized ratio (INR) was 1.2, d-dimer and fibrin degradation products were negative. She was transfused with 4 units of platelet concentrate. Her condition further deteriorated requiring escalation of inotropic support to noradrenaline 0.4 *μ*g/kg/min, dopamine 10 *μ*g/kg/min. A final diagnosis of acute respiratory distress syndrome (ARDS) and dengue hemorrhagic shock was made. Viral markers for HCV, HBV, and HIV were negative. Electrocardiogram revealed sinus tachycardia with peaked P wave. Echocardiography revealed ejection fraction of 45%, mild left ventricular global hypokinesia, mild tricuspid regurgitation with a pressure gradient of 30 mm Hg. Serum cardiac enzymes: creatine phosphokinase MB levels were 116 I.U/L (normal up to 20) and lactic acid dehydrogenase was 2489 u/L (normal up to 260). Tropnin-T was negative. An additional diagnosis of viral myocarditis was made. Dobutamine was started and noradrenaline was tapered off and subsequently stopped. Patient required an FiO_2_ of 0.8–1 to maintain of SpO_2_ > 90%. So, patient received intermittent prone ventilation to keep FiO_2_ less than 0.6% for first three days (6–8 hours each day). Central venous pressure (CVP) of 6 to 8 cm of H_2_O was maintained as further increase in CVP manifested in pulmonary edema twice. Patient had an episode of pulseless ventricular tachycardia which was managed with electrical therapy once and subsequently required 300 mg bolus amiodarone followed by 45 mg/hr infusion. By third day, patient platelet count improved and reached 66,000 and then 1,20,000/mm^3^ by 5th day. But a continuous falling trend of leucocyte count was observed from 9800 on first day to 800/mm^3^ by 5th day. She also had persistent fever of 102 to 104°F, despite being given coverage of broad-spectrum antibiotics and persistently negative cultures report (blood, urine, tracheal aspirate, CSF). When her leucocyte count dropped to less than 4000, intravenous hydrocortisone 50 mg BD was started. Despite this her leucocyte count dropped to 800/mm^3^ with differential count of polymorpholeucocytes of 46% and fever persisted. On 6th day, after written and informed consent from patients guardian, a trial of intravenous filgrastim 150 *μ*g was given for two days. Her leucocyte counts improved to 9000/mm^3^ on seventh day and 15,000/mm^3^ on eighth day. Platelet count was 1.5 × 10^3^/mm^3^. Clinically, her fever also subsided. She was weaned off from ventilator and inotropic support within next three days. Patient shifted to high dependency unit on 9th day. She was discharged uneventfully on 15th day with advice to follow-up in medical clinics.

## 3. Discussion

Our patient has persistent deterioration in clinical status and did not respond to routine management including antibiotics and mechanical ventilation support. She required inotropic support and had features suggestive of sepsis. Filgrastim has been mentioned as rescue drug in certain high risk patients with established febrile neutropenia seen in pneumonia, hypotension, sepsis syndrome, multiorgan dysfunction, fungal infection, uncontrolled primary disease, or profound neutropenia (absolute neutrophil count (ANC) <100/mcL) [4]. Filgrastim is a hematopoietic hormone that promotes the growth and maturation of myeloid cells, and particularly, the proliferation and differentiation of neutrophils [5, 6]. The usual dose of filgrastim in cancer chemotherapy is 5 *μ*g/kg/day and the duration depend on indication. But no recommendation has been made in favour or against any regimen [4]. In the absence of recommendation regarding the dose and duration of the filgrastim in such patients, we started the drug at a lower dose and duration was guided by the leucocyte response. After 2 days, when we achieved acceptable neutrophil count and clinical condition also showed an improvement, we decided to stop the drug further. Though we were apprehensive about the deterioration in lung condition as neutrophils has been proposed to be an important pathological mediator for initiating ARDS, filgrastim has been safely used in ARDS and MODS [3]. Filgrastim use decreased incidence of DIC and ARDS in adults with community-acquired pneumonia and reduced mortality from 30% to 10% in neonatal sepsis [7, 8]. 

There is evidence of immune suppression together with activation of immune-mediated destruction of target cells in DHF [1, 2]. In our case filgrastim use resulted in resolution of patient's fever and improvement of her clinical condition probably by reversing bone marrow depression and increasing leucocyte and lymphocyte count, thus enabling her to mount an immune response and leading thereby to resolution of fever. Noninfectious causes of fever including antibiotic-induced fever were ruled out as our patient temperature was persistently ≥102°F, and febrile neutropenia is an indication to continue with broad-spectrum antibiotics. Antibiotic-induced granulocytopenia was ruled out because increased ratio of polymorphonuclear leucocytes over lymphocytes as seen in sepsis was present in contrast to antibiotic-induced granulocytopenia where lymphocytes are only remaining markers of leuckocytes [9].

There is an ongoing debate over use of filgrastim as an adjunct to antibiotic therapy because of its high cost and considering its unconvincing outcomes. Safety profile and low-dose use of filgrastim for shorter duration resulted in improved outcome of our case. Further randomized controlled trials are needed in scenario of febrile neutropenia to study adjunctive role of low-dose filgrastim as rescue therapy with antibiotics especially in dengue syndrome where one of the target organ is haematopoietic system.

## References

[1] Leong ASY, Wong KT, Leong TYM, Tan PH, Wannakrairot P (2007). The pathology of dengue hemorrhagic fever. *Seminars in Diagnostic Pathology*.

[2] Lei HY, Yeh TM, Liu HS, Lin YS, Chen SH, Liu CC (2001). Immunopathogenesis of dengue virus infection. *Journal of Biomedical Science*.

[3] Wunderink RG, Leeper K, Schein R (2001). Filgrastim in patients with pneumonia and severe sepsis or septic shock. *Chest*.

[4] Smith TJ, Khatcheressian J, Lyman GH (2006). Update of recommendations for the use of white blood cell growth factors: an evidence-based clinical practice guideline. *Journal of Clinical Oncology*.

[5] Valley AW (2002). New treatment options for managing chemotherapy-induced neutropenia. *American Journal of Health-System Pharmacy*.

[6] Saab YB, Sharaf L, Zeidan I (2003). Filgrastim use: evaluation in cancer and critically ill non- cancer patients. *Cancer Therapy*.

[7] Nelson S, Belknap SM, Carlson RW (1998). A randomized controlled trial of Filgrastim as an adjunct to antibiotics for treatment of hospitalized patients with community-acquired pneumonia. *Journal of Infectious Diseases*.

[8] Bilgin K, Yaramiş A, Haspolat K, Taş MA, Günbey S, Derman O (2001). A randomized trial of granulocyte-macrophage colony-stimulating factor in neonates with sepsis and neutropenia. *Pediatrics*.

[9] Pisciotta AV (1993). Agranulocytosis during antibiotic therapy: drug sensitivity or sepsis?. *American Journal of Hematology*.

